# Exchange Rate Pass-Through, Monetary Policy, and Real Exchange Rates: Iceland and the 2008 Crisis

**DOI:** 10.1007/s11079-021-09627-5

**Published:** 2022-01-10

**Authors:** Sebastian Edwards, Luis Cabezas

**Affiliations:** University of California, Los Angeles, CA USA

**Keywords:** Exchange rates, Pass-through, Inflation, Monetary policy, Real exchange rate, E31, E52, E58, F3, F41

## Abstract

We use detailed data for Iceland to examine two often-neglected aspects of the *exchange rate pass-through* problem. First, we investigate whether the pass-through coefficient varies with the degree of *international tradability* of goods. Second, we analyze if the pass-through coefficient depends on the monetary policy framework. We consider 12 disaggregated price indexes in Iceland for 2003–2019, a period that includes Iceland’s banking and currency crisis of 2008. We find that the pass-through declined around the time Iceland reformed its *flexible inflation targeting*, and that the coefficients are significantly higher for tradable than for nontradables.

## Introduction

The nominal exchange rate plays a dual role in the macroeconomic adjustment process. On the one hand, it is at the center of the transmission mechanism of monetary policy. On the other hand, changes in the nominal exchange rate help accommodate shocks (both external and domestic) through changes in the *real* exchange rate (RER). These two roles are particularly important in (very) open economies with *inflation targeting* regimes. For the exchange rate to play these two roles effectively, the *pass-through* needs to be different for different types of goods: it has to be (significantly) higher for tradables than for nontradables. If the pass-through is similar (or the same) for both categories of goods, changes in the nominal exchange rate will *not* be translated into adjustments in the real exchange rate, and the country will face a costlier external adjustment process.^1^ This situation may arise even if the nominal exchange rate is an effective channel for transmitting monetary policy, and even if the pass-through coefficient is very low.^2^ Historically, in a number of countries with relatively high inflation the pass-through has been very high (close to 1.0) and has affected nontradable goods as much as tradable goods. This has been especially the case in Latin America.^3^

Most studies of exchange rate pass-through (ERPT) have focused on aggregate data, and have tended to ignore these two functions of the exchange rate. In fact, very few studies have concentrated on the implications of the pass-through for external adjustment and real exchange rate accommodation. Forbes et al. (2018) have pointed out, that many central banks – including some in the most advanced countries – do not rely on systematic quantitative studies of *pass-through* when considering the effects of monetary policy actions. Instead, they base their analyses on rough rules of thumb. According to Forbes et al. (2018, p.5):

“[I]n the US a 10%-dollar appreciation has been estimated to result in a fall in US consumer prices of around 0.5% (equivalent to a pass-through coefficient of 5%). In the UK, the Bank of England (BoE) has used estimates of the pass-through from exchange rate movements to UK consumer prices of around 20% to 30%.”

A number of studies for Iceland have found an exchange rate pass-through coefficient of roughly 0.4, higher than the coefficient used for policy design and evaluation in many advanced nations. (See the discussion in Section 4 below).

In this paper we use detailed data for Iceland to analyze a number of often neglected aspects of exchange rate pass-through. In particular, we investigate how changes in the nominal exchange rate are transmitted into different components of the consumer price index. Our analysis is very granular, and in the baseline estimates (Section 4) we distinguish between 12 sub-indexes in Iceland’s CPI. In Section 6 we consider even more disaggregated data, and report results for 65 indexes that go from highly tradable goods to nontradable services. This detailed analysis allows us to understand whether in Iceland the exchange rate has efficiently performed its two roles during the last 20 years or so. More specifically, we are able to rank goods by the size of the pass-through coefficient, from highest to lowest. In addition, we investigate whether there have been changes in the magnitude of the pass-through coefficient during the last 20 years. We focus, in particular, on two points in time: Iceland’s financial crisis of 2008, and the adoption of a *flexible inflation targeting* regime by the Central Bank of Iceland in 2009–2010. Following Sargent (1982), one would expect if there is a *credible* change in the *monetary regime*, the exchange rate pass-through coefficients would change in a significant way; these changes may even take place before the official (or legal) change in the monetary regime.^4^

Because of its recent history, Iceland provides an ideal case study for investigating in great detail issues related to exchange rate pass-through. Of particular interest is the fact that in 2008 Iceland faced a dramatic banking and currency crises, which was tackled successfully using somewhat heterodox tools, including capital controls. (Edwards 2018).

One of the consequences of the crisis was that in 2009–10 the Bank of Iceland introduced important changes to the inflation targeting framework that had been in place since 2001. Between that year and 2008, a rigid inflation target of 2.5% was in place, the *króna* was allowed to float freely, and there were no capital controls. The Central Bank of Iceland’s main policy tool was the short term interbank interest rate. However, this monetary regime did not work properly. Real interest rates were high, the *króna* depreciated rapidly, the inflation target was overshot every year after 2004, and the exchange rate was extremely volatile. Economic conditions between 2001 and 2008 were aptly summarized by the International Monetary Fund’s Article IV Consultation report: “inflation is high and expectations are unhinged.” (International Monetary Fund 2008, p. 24),

The 2009–2010 reforms were wide-ranging and affected governance, transparency, communications, and operating procedures. The new framework was called *Inflation Targeting Plus*. At the governance level, the three-member Board of Governors was replaced by a single Governor, and a five-member Monetary Policy Committee (MPC) with two external members. Under the new procedures, MPC decisions are announced the same day at a press conference, and the minutes of the meetings are released two weeks later. MPC members testify publicly in parliament twice a year, where they present their views on the economy and monetary policy. As Pétursson (2019, p. 375) has pointed out, this was a significant change relative to historical practices: “until 2009 minutes of monetary policy meetings were not published and information on the voting and individual views of the three-person decision-making body was not available.” At the operational level, the 2009–10 *Inflation Targeting Plus* framework introduced flexibility in the target, recognized the exchange rate as one of the most important transmission mechanisms for monetary policy, and adopted a managed float exchange rate policy. An enhanced system of macro prudential regulations that followed the guidance of the Bank of International Settlements was put in place, and capital controls on inflows were enacted as temporary measures aimed at managing capital movements and reducing exchange rate volatility. (Central Bank of Iceland 2017; Pétursson 2019; Edwards 2018, 2019).

Our main findings may be summarized as follows: the pass-through into headline inflation is significantly lower than most previous works on Iceland have estimated/considered. We find a short run ERPT of the order of 0.15 and a long term ERPT of approximately 0.23. We also found that the ERPT is different for different sectors and that there was a structural break (with a significant ERPT drop) just prior to the strengthening of the *flexible inflation targeting* framework, in 2009–2010.

The rest of this paper is organized as follows: In Section 2 we present a brief historical background of monetary and exchange rate policies in Iceland. We also provide some basic and preliminary analysis of the data. In Section 3 we develop a minimalist model to illustrate the dual role of the exchange rate in the adjustment process. In Section 4 we present the empirical results of our pass-through analysis. We report estimates using instrumental variables on error correction equations, and VARs for 12 price indexes. In Section 5 we address the question of structural breaks in the pass-through coefficients around the time the country adopted *inflation targeting plus* (2009–2010). In Section 6 we deal with extensions and robustness analysis. In this Section we present estimates of the pass-through coefficient for 65 very disaggregated sub-indexes of the CPI. Section 7 contains concluding remarks. In the Working Paper version of this article we include two appendixes where we report results for unit root tests and for alternative specifications for the error correction equations; see Edwards and Cabezas (2021).

## Iceland’s Monetary and exchange rate Policies: Historical Background and Preliminary Analysis of the Data

This Section is divided into three parts: historical developments in Iceland‘s exchange rate regime between 1918 and 2010; post 2010 issues related to monetary policy, exchange rates, and pass-through; and an analysis of the basic data on inflation and exchange rates.^5^

### 1918–2010

Iceland obtained monetary autonomy by the end of World War I, when it achieved sovereignty from Denmark in 1918.^6^ In 1920 the country faced the first of many currency crises. Icelanders responded to it by implementing currency controls. Two years later, in 1922, a 23% devaluation of the *króna* followed. Between 1925 and 1939 the *króna* was pegged to the British pound; from 1939 to 1949 it was pegged to the US dollar. In 1931, at the time the United Kingdom abandoned the gold standard and devalued sterling, Iceland once again resorted to exchange controls; their intensity varied through the years, becoming more stringent during times of crises and during World War II. In 1970 Iceland became a member of the European Free Trade Association (EFTA), and in 1994 it joined the European Economic Area (EEA). At the time, and as a condition for joining the EEA, Iceland was required to lift exchange controls. This situation would last until 2008, when the most recent crisis erupted, at which time controls were reintroduced.^7^

In 1971, after the collapse of the Bretton Woods system, Iceland adopted an adjustable peg with respect to the USD; this policy lasted until 1974. Between that time and 1983, the degree of flexibility of the exchange rate was somewhat increased, and the country followed what the Central Bank of Iceland called a “managed float” policy aimed at targeting the exchange rate. At first it was targeted relative to the USD and then relative to various currency baskets.

Between 1984 and 1989 the exchange rate policy became more rigid. However, since inflation didn’t subside, small devaluations – ten overall – were implemented; for all practical purposes the country was following a variation of a “crawling peg” regime. Between 1990 and 1995 a renewed effort at exchange rate stability was made, and several exchange rate bands were used. Initially, the reference point was given by a 17 currencies basket, and the band width was ± 2.25% relative to the benchmark. The basket was redefined in 1992; the USD was given an 18% weight, the Japanese yen 6%, and the ECU 76%.

The *króna* was devalued in 1992 and 1993. In 1996 the width of the band was increased to ± 6%, and a new basket of 16 currencies was defined. From 1996 through 2000 the currency was allowed to move freely within the band; in February 2000 the band was once again widened, this time to ± 9% relative to the basket target. In 2001 the exchange rate target – or target zone – was eliminated and an inflation target was adopted. This regime lasted until the 2008 crisis.

**Fig. 1:**
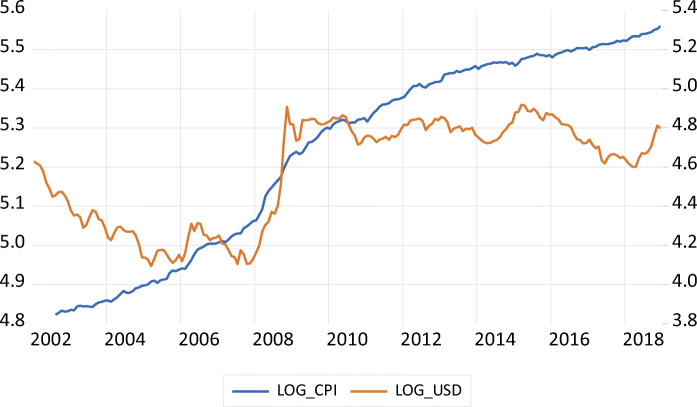
Log of CPI (in the left-axis) and bilateral exchange rate (in the right-axis) with respect to the US Dollar, 2001–2019

In 2003 Iceland privatized its banking system. What followed was a credit boom. Between 2004 and 2008, most Icelandic banks financed this large expansion of credit with foreign funds. As Raza and Zoega (2019) have documented, during this period the assets of the largest banks grew between 50% and 60% annually. The results were an impressive asset and housing price bubble, and a current account deficit that grew to an astounding 20% of GDP. A collateral effect of this situation was that the *króna* appreciated with respect to the US dollar by almost 20% (see Fig. 1).

In 2008 Iceland was subject to a classical “sudden stop.” In a matter of a few months – a few weeks, really – capital flowing into the country stopped. This situation was followed by a major macroeconomic crisis characterized by a significant “current account reversal,” a steep depreciation of the currency (over 50% with respect to the US dollar), a jump in unemployment, an acceleration of inflation, massive bank failures, and significant bankruptcies.^8^

### 2010–2020

After the October 2008 banking and currency crisis, Iceland undertook a massive adjustment program that included heterodox components such as stiff capital controls. Banks had to be rescued by the government. Eight years after an almost complete meltdown, the recovery was practically complete (Aliber and Zoega 2019). In early 2017 Gross Domestic Product was nearly back to its trend, inflation was below 2% per annum, unemployment was 1.5%, and there was a sizeable current account surplus (approximately 6% of GDP). Many of the emergency measures undertaken in the aftermath of the 2008 crisis were dismantled.^9^

In September 2017 the CBI released a report, titled “*Monetary policy based on inflation targeting: Iceland’s experience since 2001 and post-crisis changes*.” In it the Central Bank argued that the rapid recovery of the economy was, to a large extent, the result of a change in monetary policy in 2010. According to the CBI, while monetary policy was ineffective during the period 2001–2008, it greatly improved in terms of efficiency and effectiveness since 2010–2012. One of the objectives of this paper, as noted earlier, is to analyze whether this alleged structural and discreet improvement in monetary policy was reflected in reductions in the exchange rate pass-through coefficients.

### Preliminary Analysis of the Data

**Fig. 2:**
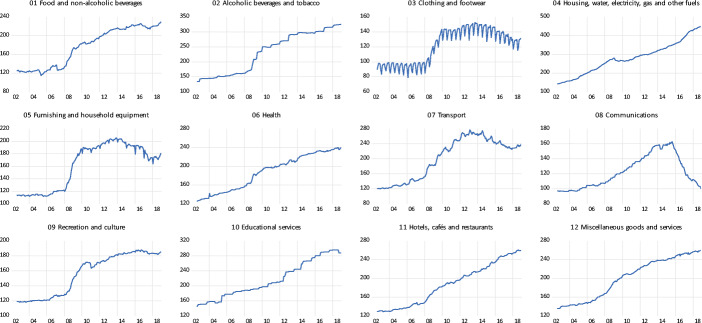
Consumer Price Indexes for Twelve Goods Categories in Iceland: 2002–2019

Figures 1 and 2 summarize the data used in this paper. In Fig. 1 we show the evolution of the log of the CPI and the log of an index of the *króna*/USD exchange rate between 2001 and 2019. An increase in the exchange rate index denotes an increase in the price of the USD, or a depreciation of the domestic currency. The 2008 crisis is visible in this figure: we can see the jump in the price of the dollar after 2008. It is also possible to see the acceleration of inflation just before the 2008 crisis. In 2004 and 2005 inflation was 3.2% and 4.0%. In 2008 and 2009 it was 12.4% and 12.0%. By 2017 it had declined to 1.8%.

Figure 2 presents, for 2002–2019, the evolution of 12 sub-indexes of the CPI. These data are in levels, and are not seasonally adjusted. As may be seen, different indexes show different behavior. In some categories the effect of the 2008 devaluation is quite clear, while in others it is almost imperceptible. The 12 price categories are:

1. Food and non-alcoholic beverages;
2. Alcoholic beverages and tobacco;
3. Clothing and footwear;
4. Housing, water, electricity, gas and other fuels;
5. Furnishing and household equipment;
6. Health;
7. Transport;
8. Communications;
9. Recreation and culture;
10. Educational services;
11. Hotels, cafés and restaurants;
12. Miscellaneous goods and services.

Some of these categories have a heavy component (or are fully comprised) of nontradables (i.e. educational services), while other are dominated by tradables (i.e. clothing and footwear). In the rest of this paper categories 1, 2, 3, 5, and 7 will be classified as being (mostly) tradables.^10^ On the other hand, categories 4, 6, 9, 10 and 11 are (mostly) nontradable. This leaves two groups unclassified: Communications and Miscellaneous goods and services. In the rest of the analysis we consider them (generally) being nontradables. However, and as is noted below, the overall results and the main message of the paper are not affected in a significant way if they are excluded from the calculations or if they are classified as tradable.

To summarize, an important objective of our analysis is to determine whether these different categories have different pass-through coefficients, and whether these can be ranked from higher to lower depending on the degree of tradability of the category in question.

## The Analytics of exchange rate Pass-through: A Minimalist Model

In this Section we present a minimalist model of a small open economy that captures some of the most salient issues discussed above. Although the key equations are not formally derived from an optimization process, the main building blocks are consistent with intertemporal optimizing models recently used to study pass-through issues (Forbes et al. 2018; Corsetti et al. 2008).^11^ Eq. (1) is the price level (CPI), which has two components: the domestic prices of tradable and nontradable goods. Since this is an index at the consumer level, it is expected that the share of nontradables is quite high. Equation (2) is the real exchange rate, defined as the domestic relative price of tradables to nontradables. Equation (3) defines the price of tradables as a Cobb-Douglas index of the domestic prices of importables and exportables. Equation (4) is the domestic price of importables. This is a very general representation, which can accommodate either “pricing to market,” or a strict version of the “law of one price.” In this equation, μ is the markup, and is defined in eq. (5), where η is the demand elasticity for imports in the (small) home country.^12^ The coefficient *ϕ* is a factor of proportionality that captures possible (additional) deviations from goods’ arbitrage. *E* is the nominal exchange rate, and is one of the variables of interest in this analysis; the other one is the price level, as defined in eq. (1). Notice, from eq. (4), that if *ϕ* = 1, and *η* =  − ∞, then the domestic price of importables will be equal to their world price multiplied by the nominal exchange rate. That is, the law of one price would hold. In the more general version of the model it is possible to think that both μ and *ϕ* are functions of the nominal exchange rate *E*.^13^ See the discussion below.

1 $$ {P}_t={P}_{T,t}^{\alpha }\ {P}_{N,t}^{\left(1-\alpha \right)} $$

2 $$ {\rho}_t=\frac{P_{T,t}}{P_{N,t}} $$

3 $$ {P}_{T,t}={P}_{M,t}^{\beta }\ {P}_{X,t}^{\left(1-\beta \right)} $$

4 $$ {P}_M=\mu\ {\left({E}_t{P}_M^{\ast}\right)}^{\phi } $$

5 $$ \mu =\frac{\eta }{1+\eta };\kern2.25em \eta <-1 $$

Equation (6) is the domestic price of exportables, where *φ* is a factor of proportionality. If *φ* = 1, then exports are subject to the law of one price. Equation (7) defines the terms of trade as the ratio of the price of exportables to importables.

6 $$ {P}_X={\left({E}_t{P}_X^{\ast}\right)}^{\varphi } $$

7 $$ {\tau}_t=\raisebox{1ex}{${P}_{X,t}$}\!\left/ \!\raisebox{-1ex}{${P}_{M,t}$}\right. $$

8 $$ {N}_t^D\left({\rho}_t,{r}_t,{Z}_t\right)={N}_t^S\left(\frac{W_t}{P_t^N},{V}_t\right) $$

Equation (8) is the equilibrium in the nontradables market. The demand for nontradables, ${N}_t^D$, depends on its relative price or real exchange rate, *ρ*_*t*_, the central-bank monetary policy interest rate *r*_*t*_, and a variable that measures other aspects of macroeconomic policy including the country’s fiscal stance, *Z*_*t*_. The supply of nontradables, in turn, depends on the real wage rate in the nontradables sector $\left(\frac{W_t}{P_t^N}\right)$, and on a productivity parameter *V*_*t*_; *W*_*t*_ is the nominal wage rate, in domestic currency. Equation (9), specifies wages’ adjustments.

9 $$ \hat{W_t}=\gamma\ {\pi}_t^e+\psi\ {\pi}^T+\delta \kern0.50em {\pi}_{t-1}+{s}_t $$

Where $\hat{W_t}$ is the rate of change in nominal wages, ${\pi}_t^e$ is future expected inflation, as of period *t*, and *π*^*T*^ is the central bank’s inflation target. This expression states that every period wages are updated by a “catch up” term, given by past inflation, *π*_*t* − 1_, and by a combination of expected inflation and the inflation target. For credible central banks ${\pi}_t^e={\pi}^T$. Under most circumstances (*γ* + *ψ*) ≤ 1. Coefficient *δ* captures the role of backward looking adjustments. Stability requires that *δ* < 1.^14^ In what follows it is assumed that expected inflation is equal to current inflation plus an error term *h*_*t*_ with the usual properties (zero mean and finite variance): ${\pi}_t^e={\pi}_t+{h}_t$.

The coefficient *γ* defines how important expected inflation is in the wage setting process. A high value of *γ*, with a low value of *ψ* reflects *a low degree of credibility of central bank policy*. A credible stabilization program will be characterized by a very low (possibly zero) *γ* and a high (possibly equal to one) *ψ*. The term *s*_*t*_ is a productivity shock.

Equations (1) through (9) provide a minimal structure for analyzing the mechanics of pass-through, or the connection between changes in the nominal exchange rate (${\hat{E}}_t$), domestic inflation ( *π*_*t*_), and real exchange rate changes ($\hat{\rho}$). In extensions sketched below, we consider a more complete formulation that includes expressions for the evolution of the nominal exchange rates and a monetary policy rule in the spirit of the Taylor Rule.

In order to focus on the pass-through issue, we make some simplifying assumptions, which can be easily relaxed (see below for details and extensions). First, we assume that there are no changes in the terms of trade, in policy variables, international prices, or productivity. We further assume that *ϕ* = *φ* , and that *η* =  − ∞. In addition, and without loss of generality, we assume that the central bank’s inflation target is equal to zero, *π*^*T*^ = 0, and, as noted, that the error term *h*_*t*_ has a zero mean.

The solutions for nontradable inflation$, {\pi}_t^N$; CPI inflation, *π*_*t*_; and the change in the real exchange rate $\hat{\rho_t},$are given by eqs. (10)–(12):

10 $$ {\pi}_t^N=\phi\ \left(\frac{\lambda_1-{\lambda}_2\ \gamma\ \alpha }{\lambda_1-{\lambda}_2\left(1-\gamma \left(1-\alpha \right)\right)}\right)\ \hat{E_t}-\left(\frac{\lambda_2\ \delta }{\lambda_1-{\lambda}_2\left(1-\gamma \left(1-\alpha \right)\right)}\right)\ {\pi}_{t-1}. $$

11 $$ {\pi}_t=\left\{\left(1-\alpha \right)\phi \left(\frac{\lambda_1-{\lambda}_2\ \gamma\ \alpha }{\lambda_1-{\lambda}_2\left(1-\gamma \left(1-\alpha \right)\right)}\right)+\phi\ \alpha \right\}\ \hat{E_t}-\left(\frac{\left(1-\alpha \right){\lambda}_2\ \delta }{\lambda_1-{\lambda}_2\left(1-\gamma \left(1-\alpha \right)\right)}\right)\ {\pi}_{t-1}. $$

12 $$ \hat{\rho_t}=\phi\ \left\{1-\left(\frac{\lambda_1-{\lambda}_2\ \gamma\ \alpha }{\lambda_1-{\lambda}_2\left(1-\gamma \left(1-\alpha \right)\right)}\right)\ \right\}\hat{E_t}+\left(\frac{\lambda_2\ \delta }{\lambda_1-{\lambda}_2\left(1-\gamma \left(1-\alpha \right)\right)}\right)\ {\pi}_{t-1}. $$

In eqs. (10)–(12), *λ*_1_ ≥ 0 is the price elasticity of demand of nontradables, relative to the real exchange rate; *λ*_2_ ≤ 0 is the elasticity of supply of nontradable with respect to real wages.

The term with lagged inflation indicates that in this setting there is inflationary inertia or persistence. This arises because of the backward-looking component in the wage adjustment equation. If the wage setting mechanism is fully forward-looking, then, *δ* = 0. In this case there is no lagged inflation term, and no inertia in eq. (11). An interesting case emerges when wages are fully indexed to past inflation, and the exchange rate follows a crawling peg rule with 100% adjustment according to past inflation. This combination, which at some time was popular in some Latin American countries such as Brazil, results in a loss of the inflation anchor. In this case inflation becomes a random walk.^15^

In eq. (11), the short term pass-through coefficient -- the parameter of interest in this study --, is given by:

13 $$ Short\ term\ ERPT=\left\{\left(1-\alpha \right)\phi \left(\frac{\lambda_1-{\lambda}_2\ \gamma\ \alpha }{\lambda_1-{\lambda}_2\left(1-\gamma \left(1-\alpha \right)\right)}\right)+\phi\ \alpha \right\}. $$

As may be seen, this coefficient depends on a number of factors: the weight of tradables and non tradables in the price index, the elasticities of demand and supply in the nontradable market, the coefficient of deviation from the law of one price in tradables *ϕ*, and the importance of expected future inflation in the wage setting equation *γ*. As noted above, this coefficient *γ* can be interpreted as reflecting the degree of credibility of monetary policy. If the central bank inflation target is credible, the γ will be low, possibly zero.

According to eq. (13), a higher γ results in a higher pass-through, as does a higher weight for tradables in the CPI. The pass-through will also increase with a more inelastic supply of nontradables and a more elastic demand for those goods. As noted, one of the goals of this paper is to estimate a pass-through coefficient for a number of components of the CPI (12 and 65). In Section 6, and as a test for the reasonability of the model, we report results from using data for import prices for 7 categories, in Iceland.

The model can be expanded in several ways. For example, an explicit equation may be written for the dynamics of the nominal exchange rate -- eq. (14). The currency depreciates (appreciates) if the equilibrium real exchange rate $\overset{\sim }{\rho }$ exceeds (is below) the actual RER in the previous period. This is a gradual process, with the speed of adjustment given by the parameter *θ*. In addition, the nominal exchange rate responds, through the carry trade, to changes in the monetary policy interest rate. In this analysis, and in order to simplify the presentation, we can assume that the equilibrium real exchange rate $\overset{\sim }{\rho }$ is exogenous. In a more complete setting, it is possible to endogenize it, and assume that it depends on the relation between the sustainable level of Net International Investment Position (NIIP) and other “fundamental” variables. Another extension is to consider an explicit Taylor rule, as in eq. (15).

14 $$ {\hat{E}}_t=\theta\ \left(\overset{\sim }{\rho }-{\rho}_{t-1}\right)-\xi\ d{r}_t $$

15 $$ {r}_t={\delta}_0\ \left({\pi}_t-{\pi}^T\right)+{\delta}_1\ (gap);{\delta}_0\ge 1;{\delta}_1\ge 0 $$

From an extended model that includes (14) and (15), it is possible to derive an equation equivalent to (11), with an explicit pass-through coefficient that would depend on the parameters discussed above, as well as on *θ*, *δ*_0_, and *δ*_1_. In this case, of course, the exchange rate becomes endogenous, a fact that is taken into account in the estimations reported in Sections 4, 5 and 6 below.

## Empirical Results: Baseline Estimates

Our empirical strategy consists of analyzing the problem from different perspectives, and using different techniques. The goal is to produce a consistent, robust, and persuasive body of evidence on the pass-through issue. More specifically, we report results from two estimation methods:

- Instrumental variables to estimate error correction equations;
- Structural VARs.

(OLS results are available on request.)

### Error Correction Equations: Instrumental Variables

We estimated a number of error correction equations for the CPI and its 12 sub-indexes, using quarterly data from 2003 through the first quarter of 2019. The baseline specification has the following form:

16 $$ {\pi}_t={\alpha}_0+{\alpha}_1\ \hat{E_t}+{\alpha}_2\ {\pi}_{t-1}+{\alpha}_3\ \Delta {\pi}_{t-1}+{\beta}_1\ {z}_t+{\omega}_{t.} $$

*π*_*t*_ is inflation, $\hat{E_t}$ is the rate of change of the *króna* relative to a basket of currencies (an increase is a depreciation of the krona), the *z*_*t*_ are other covariates, including international inflation, *π*^∗^. This specification allows us to distinguish between the short-run ERPT (coefficient *α*_1_) and the long-run effect, given by $\frac{\alpha_1}{\left(1-{\alpha}_2\right)}$. *ω*_*t*_ is a normally distributed i.i.d. disturbance. Notice that this expression allows for richer dynamics than the one derived in the model. This is reflected by the inclusion of the term Δ*π*_*t* − 1_ in the equation. Our identifying strategy is based on the fact that in a small country, such as Iceland, world interest rates, and changes in the international terms of trade are valid instruments; they affect the exchange rate, but are not impacted by Icelandic inflation. We also use quarterly dummies as instruments.

Before reporting the results, a few words on previous studies: Historically, the accepted view was that the ERPT in Iceland (for headline CPI inflation) was close to 0.40. For example, Pétursson (2002) estimates a price-wage system and finds a long-run coefficient on import prices of 0.40, using data from 1973 to 1999. This was confirmed by additional research using cross-country data and undertaken just before the crisis: using data from 1985 to 2005, Pétursson (2008) found an ERPT into headline inflation equal to 0.43. A study by Ólafsson et al. (2011), based on surveys, confirmed these results. Pétursson (2010) estimated pass-through equations using a cross-country sample, and investigated whether there were structural beaks. No breaks were found during the 1985–2005 period. In September 2017, the CBI reported new evidence. In page 25 of the report we read: “[C] urrency appreciation also helps to keep domestic inflationary pressures under control, and furthermore, it directly reduces inflation through lower import inflation. In the same manner, currency depreciation can mitigate a downturn. Without exchange rate flexibility, business cycles could become more volatile, as an important part of the economy’s shock absorbing capacity has been removed and an important channel for monetary policy transmission to the real economy has been closed off.” In this report, however, there are no estimates for pass-through coefficients. In a recent paper, Pétursson (2020) used data for 2003–2016, to estimate a Phillips Curve and found an ERPT onto import prices of 0.20, about one half of what was estimated in the early 2000s. As will be seen, this estimate is in line with our own findings reported in Sections 4 and 5 of this paper.

In Table 1 we present the baseline results for the IV estimation of equations of the type of (16) for CPI inflation and its 12 main components.^16^ The first column refers to the percentage change in the aggregate price level (CPI). The columns that follow are for the disaggregated sub-indexes. Results obtained with alternative specifications are presented in Edwards and Cabezas (2021) in the Appendix C. Tests for weak exogeneity indicate that the choice of instruments is appropriate.^17^ The results may be summarized as follows:

- There are significant differences in the ERPT coefficients across the different components of the CPI. They range, in the short run, from almost 40% (Clothing and footwear, a highly tradable sector) to basically zero for Educational services, which is essentially a nontradable service.

**Table 1:** Exchange Rate Pass-Through onto CPI and Components: Error Correction, Instrumental variables, Iceland 2003–2019

	AGGREGATE	(1)	(2)	(3)	(4)
VARIABLES	CPI	Food and non-alcoholic beverages	Alcoholic beverages and tobacco	Clothing and footwear	Housing, water, electricity, gas and other fuels
NEER (% change)	0.154**	0.176*	0.131	0.393**	0.0338
	(0.075)	(0.105)	(0.089)	(0.164)	(0.060)
Consumer Price Index (% change) = L,	0.318	0.205	0.380***	0.23	0.571***
	(0.233)	(0.185)	(0.139)	(0.228)	(0.12)
Consumer Price Index (% change) = D,L,	−0.00114	0.137	−0.158	−0.582***	−0.0248
	(0.241)	(0.16)	(0.123)	(0.121)	(0.129)
Production Price Index (foreign)	0.220**	−0.206	−0.450*	−0.361	0.309**
	(0.112)	(0.234)	(0.247)	(0.341)	(0.135)
Constant	0.00572**	0.00743**	0.00889***	0.00306	0.00576**
	(0.003)	(0.003)	(0.003)	(0.004)	(0.002)
Observations	64	64	64	64	64
R-squared	0.482	0.357	0.226	0.882	0.371
	(5)	(6)	(7)	(8)	(9)
VARIABLES	Furnishing and household equipment	Health	Transport	Communications	Recreation and culture
NEER (% change)	0.385***	0.159**	0.454***	0.0752	0.175***
	(0.131)	(0.068)	(0.128)	(0.093)	(0.050)
Consumer Price Index (% change) = L,	0.465***	−0.093	−0.0915	0.735***	0.429***
	(0.157)	(0.193)	(0.134)	(0.108)	(0.108)
Consumer Price Index (% change) = D,L,	−0.367***	0.12	0.315***	−0.246*	0.0491
	(0.134)	(0.148)	(0.118)	(0.131)	(0.121)
Production Price Index (foreign)	−0.0458	−0.343**	1.218***	0.0727	−0.327**
	(0.264)	(0.167)	(0.277)	(0.197)	(0.138)
Constant	0.00169	0.0112***	0.00303	−0.000425	0.00429**
	(0.003)	(0.003)	(0.003)	(0.002)	(0.002
Observations	64	64	64	64	64
R-squared	0.401		0.511	0.485	0.489
	(10)	(11)		(12)	
VARIABLES	Educational services	Hotels, cafés and restaurants		Miscellaneous goods and services	
NEER (% change)	−0.0612	0.0421		0.120**	
	(0.069)	(0.061)		(0.054)	
Consumer Price Index (% change) = L,	0.135	0.28		0.282	
	(0.134)	(0.194)		(0.173)	
Consumer Price Index (% change) = D, L,	0.324***	−0.0287		0.0631	
	(0.119)	(0.145)		(0.155)	
Production Price Index (foreign)	−0.2	0.058		−0.00033	
	(0.162)	(0.144)		(0.123)	
Constant	0.0104***	0.00729***		0.00627***	
	(0.002)	(0.003)		(0.002)	
Observations	64	64		64	
R-squared	0.255	0.13		0.085	

Standard errors in parentheses

*** *p* < 0.01, ** *p* < 0.05, * *p* < 0.1

In Table 2 we summarize our results, and organize the *long-run* ERPT coefficients for the different sub-indexes from lowest to highest. As may be seen, the five categories with the lowest long term pass-through correspond to nontradable sectors:

- Educational services;
- Hotels, cafés and restaurants;
- Housing, water, electricity gas and other fuels;
- Health;
- Miscellaneous goods and services.

**Table 2:** Short Run and Long Run Exchange Rate Pass-Through Coefficients for CPI and components: 2003–2019*

CATEGORIES	Short term	Long term
Educational services	−0.06	−0.069
Hotels, cafés and restaurants	0.042	0.058
Housing, water, electricity, gas and other fuels	0.033	0.077
Health	0.159	0.145
Miscellaneous goods and services	0.12	0.167
Alcoholic beverages and tobacco	0.131	0.211
Food and non-alcoholic beverages	0.176	0.221
***Aggregate CPI***	***0.154***	***0.226***
Communications	0.075	0.283
Recreation and culture	0.175	0.306
Transport	0.454	0.416
Clothing and footwear	0.393	0.51
Furnishing and household equipment	0.385	0.72

*Obtained from the error correction estimates reported above.

The two categories with the highest pass-through are comprised of heavily tradable items (Furnishing and household equipment, and Clothing and footwear). They both have long term pass-through coefficients above 50%.

The mean for the short term ERPT coefficients for *nontradable* goods in Table 2 is 0.078; the median is 0.075. The long term mean and median for ERPT for nontradables are 0.138 and 0.145, respectively.^18^

For the (mostly) tradable goods in Table 2 the mean and median ERPT are 0.308 and 0.385, in the short run. Both the long run mean and median for ERPT for tradable goods is 0.416.

Based on the mean estimates, the results in Table 2 suggest that, with other things given, a 10% depreciation of the *króna* will result in a long run increase in the relative price of tradable goods of about 4%. It will also generate a 2.3% increase in CPI inflation.

The analysis presented so far assumes that the exchange rate pass-through coefficients have been stable during the period under study. However, as noted in the introduction to this paper, it is possible that around 2009, when new monetary policy procedures – *inflation targeting plus* -- were implemented and flexible inflation targeting was adopted, there were changes in the pass-through coefficients. The question of possible “breakpoints” is investigated in Section 5. In Section 6 we analyze other extensions, including whether the pass-through depends on the nature of external shocks. In addition, we present a number of robustness tests.

### VARs

In order to investigate further the nature of exchange rate pass-through, and to check for the robustness of the results reported above, we also estimated a number of vector auto regression (VAR) models. In the estimation the following endogenous variables were included: percentage change in real GDP in Iceland, change in short-term interest rates in Iceland, percentage change in a weighted average nominal exchange rate with respect to Iceland’s main trade partners, and percentage change in the aggregate CPI and in its components. The following exogenous variables were included: percentage change in foreign prices; percentage change in GDP in the European Union; logarithm of terms of trade; change in short-term interest rates in the Euro zone.

We relied on the Hannan-Quinn information criteria to determine the lag structure. We settled for a four quarters lag model.^19^ In order to compute impulse response functions we use the inverse of the Cholesky factor of the residuals variance-covariance matrix so that innovations become uncorrelated.^20^ The short term impact is calculated using the first quarter impulse response function; the long term pass-through corresponds to the accumulated impulse response function for eight quarters. The order of the variables is: GDP growth, change in the domestic interest rates, the percentage change in the nominal exchange rate index, and inflation.

**Fig. 3:**
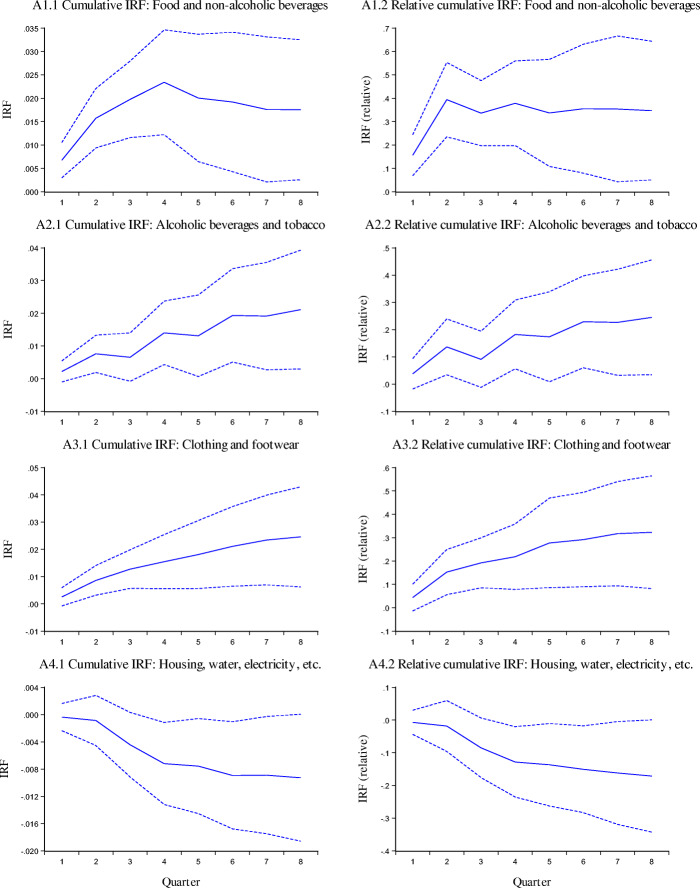

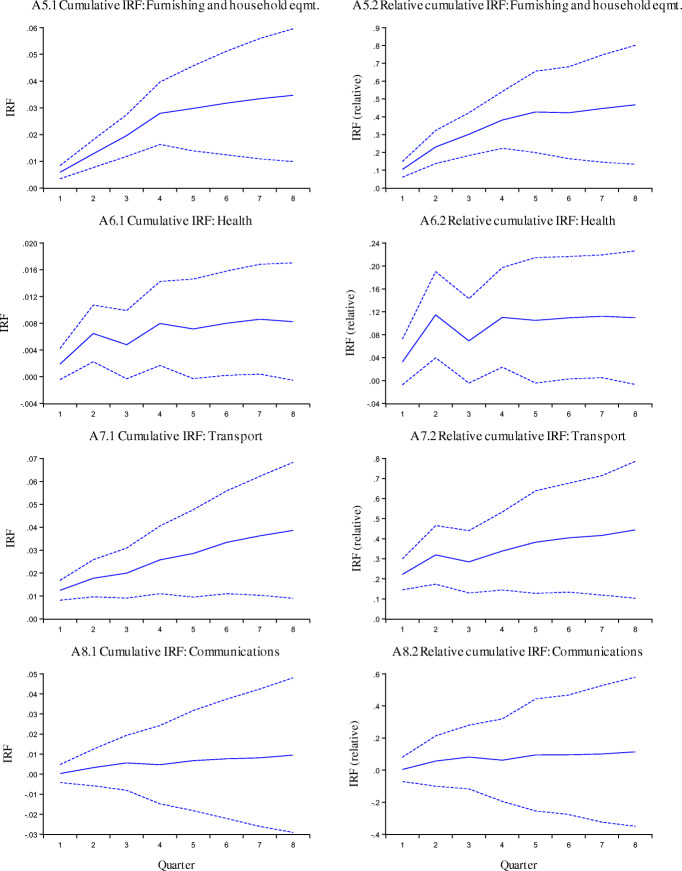

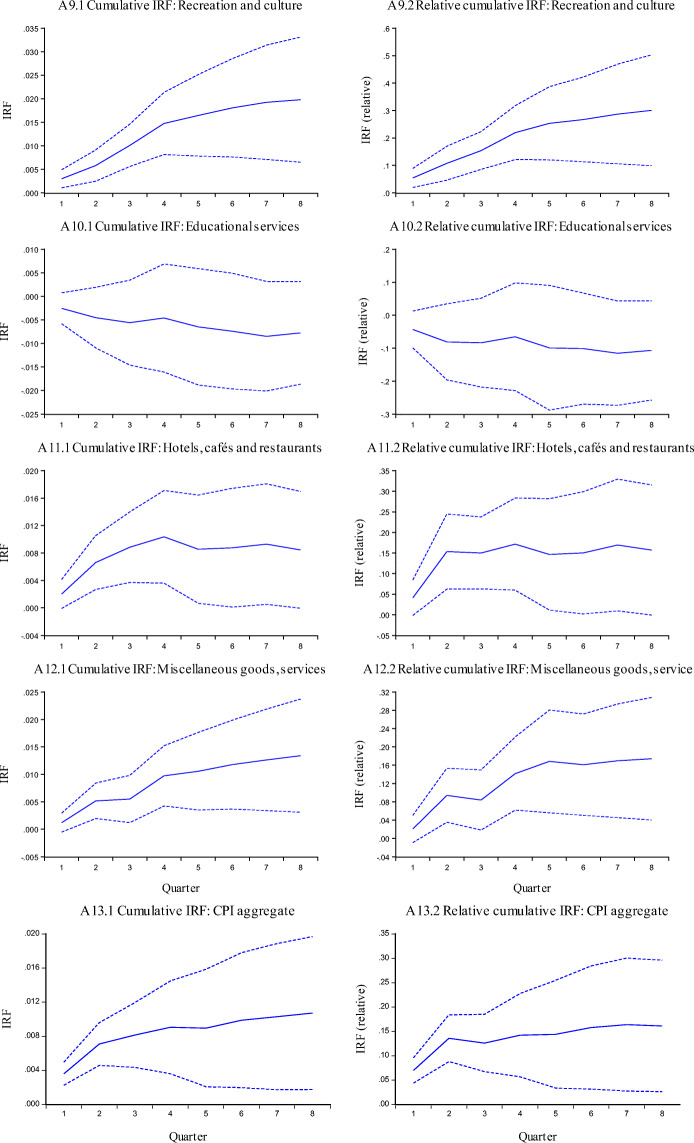
Cumulative IRF for CPI and Components, dashed lines corresponds to ±2 standard errors

The impulse response functions for CPI inflation are reported in Fig. 3. In these figures the left-hand panels correspond to the accumulated IRF for a nominal exchange rate shock. The right-hand side panels are for a relative impulse response function, defined as the accumulated response of inflation, divided by the accumulated response of the exchange rate. The first 12 panels correspond to the different sectors, and the last one to the inflation rate of the aggregate CPI.

The results obtained from this analysis are summarized in Table 3, where we present the short term and long term pass-through coefficients for the price indexes considered in this study, and their components. These results for the CPI broadly confirm those obtained with the error correction model:

1. The pass-through into the aggregate CPI is rather low.
2. There are significant differences in the pass-through for the different categories of the CPI.
3. Some nontradable sectors (i.e. education) have an ERPT that is not significantly different from zero.
4. Sectors with highest ERPT correspond to those with imported components of tradables.
5. The above indicates that the nominal exchange rate has been playing an efficient role in Iceland. It has served as a transmission mechanism for monetary policy, and it has helped accommodate external shocks through changes in the real exchange rate.

**Table 3:** Short Run and Long Run Exchange Rate Pass-Through Coefficients, VAR estimates for CPI and components: 2003–2019*

CATEGORIES	Short term	Long term
Housing, water, electricity, gas and other fuels	−0.007	−0.171
Educational services	−0.044	−0.107
Health	0.032	0.11
Communications	0.004	0.114
Hotels, cafés and restaurants	0.041	0.157
***Aggregate CPI***	***0.07***	***0.161***
Miscellaneous goods and services	0.021	0.174
Alcoholic beverages and tobacco	0.038	0.245
Recreation and culture	0.054	0.3
Clothing and footwear	0.043	0.323
Food and non-alcoholic beverages	0.156	0.347
Transport	0.222	0.444
Furnishing and household equipment	0.104	0.467

*Results obtained using the Cholesky decomposition. Estimates using SVARs are very similar. See text for details.

Having reported the broad similarities in the results from our different estimation techniques, it is interesting to notice that there are also some differences in the point estimates of the coefficients for CPI under the two methods. Indeed, the estimates from the VARs suggest lower ERPT than those from the error correction model. Take, for example, the estimates for the aggregate CPI inflation: the error correction estimate for the short run is 0.154, and it is 0.070 for the VAR. For the long run, the error correction coefficient is 0.226 and it is only 0.161 for the VAR estimates. Understanding the root cause for these differences is the topic for further research. The mean and median coefficients from the VAR analysis are as follows:

**Table:** 

	Short term impact (one quarter)	Long run impact
Mean for nontradable goods	0.014	0.082
Median for nontradable goods	0.021	0.114
Mean for tradable goods	0.113	0.365
Median for tradable goods	0.104	0.345

As in the error correction IV estimations, the coefficients for nontradables are significantly lower (both in the short and long run), than for tradables. Edwards (2007), among others, documented that this is not always the case. In particular, in a number of emerging markets in Latin America the pass-through into nontradables is very high, often undistinguishable (statistically speaking) from the pass-through to tradables. In those instances, and in contrast with the experience of Iceland, changes in the nominal exchange rate are not translated into real exchange rate adjustments.

## Breakpoints and Monetary Policy Credibility

In its 2017 report, the Central Bank of Iceland pointed out that there was an important improvement in the “quality” of monetary policy around 2010. According to the bank, at the center of the successful new monetary policy was an increase in the degree of *credibility* of the *inflation targeting plus* regime, including the change in CBI governance and communication strategy.^21^ As pointed out in Section 3, increased credibility would be reflected in a lower parameter γ in the model. This, in turn, would result in a lower pass-through coefficient for the aggregate CPI.^22^

In this section we investigate whether there have been structural breaks in the pass-through equations in Iceland during the period under study.

We use the Bai and Perron (1998) method on CPI inflation regressions to identify possible breakpoints and their dates. This approach can be used in two ways: One option is to ask the data to tell us endogenously the number of breakpoints (if any). In this case, the global optimization procedure identifies the number of breakpoints *and* their dates, and provides estimates of the coefficients in the different “regimes.” An alternative way of implementing this method is to define exogenously the number of breakpoints (but not their dates). If the null of no breakpoints is rejected, we ask the data to tell us the dates of the (possible) breakpoints. Given the small number of observations in our data set, the first and more general approach that tests for the possibility of several breakpoints is not efficient. Thus, we followed the second avenue, and tested for the presence of only one breakpoint in each series during the period under analysis.

The null of no breakpoints is rejected for the CPI and most of its components (*p*-values of the test <0.05). The identified date of the structural break is the first quarter of 2009, which corresponds to the launching of the reforms of the Central Bank of Iceland. The date, however, is somewhat earlier than the official change in monetary policy *procedures,* or what the CBI has called the adoption of *inflation targeting plus*. This suggests that the announcement of a change in policy framework was credible, in the sense described by Sargent (1982) in his classical study on stabilization. The point estimates indicate that after 2009q1 both the short and long run ERPT coefficients *declined significantly*. In Table 4 we present instrumental variables and least squares estimates with interactive dummy variables to obtain estimates of the ERPT in both regimes (pre and post 2009q1). The point estimate for the least squares short term pass-through before the change of regime is 0.129; it is only 0.050 after the break. The long term ERPT coefficient, is 0.194 in the earlier period and 0.075 in the latter period. For the IV estimate the short term ERPT declines from 0.16 to 0.052; the long term goes from 0.227 to 0.074. As noted, this supports the view that around the time of the breakpoint there was an improvement in the degree of credibility of monetary policy. The analysis also shows that there were no changes in the dynamics of inflation adjustment; that is, the coefficient of lagged inflation interacted with the dummy variable was never significant.

**Table 4:** Exchange Rate Pass-Through Coefficient on CPI and Break Points: Error Correction, Instrumental variables (IV) and Ordinary Least Squares (OLS), Iceland 2003–2019*

	(1)	(2)
VARIABLES	IV	OLS
NEER (% change)	0.160***	0.129***
	(0.0323)	(0.0192)
NEER(% change) * 1{t ≥ 2009q1}	−0.108	−0.079**
	(0.107)	(0.0351)
Consumer Price Index (% change) = L,	0.296***	0.335***
	(0.0938)	(0.0876)
Production Price Index (foreign)	0.255***	0.239***
	(0.0948)	(0.0898)
Constant	0.00549***	0.00540***
	(0.00140)	(0.00141)
Observations	64	64
R-squared	0.572	0.590

*Break point is statistically significant dated with Bai-Perron method in 2009q1. Interactive term corresponds to a dummy variable equal to 1 starting in 2009q1 until the end of the sample. Columm (1) uses instrumental variables, column (2) is OLS estimation.

Standard errors in parentheses

*** p < 0.01, **p < 0.05, * p < 0.1

We also estimated error correction equations with interactive dummy variables for the CPI’s twelve components. A summary of the results obtained from the least squares estimation are in Table 5.^23^ A summary of our findings is as follows:

- In both regimes there is a difference in ERPT across sectors. As in the results reported above, the ERPT is significantly higher for tradables than for nontradables.
- In 10 of the 12 categories there is a decline in the short term ERPT. In eight of those the drop is large and exceeds 5 percentage points.
- There is an increase in the short term ERPT in Transport and Communications. It is not clear from the data what is behind this change. An interpretation of this result requires additional research on the specific nature of these sectors.
- The results for the long term ERPT are similar to those for the short term: there is a decline in the ERPT in 10 of the 12 sectors. For long term estimates, nine of the sub-indexes exhibit a decline of 5 percentage points or more. Again, the only subgroups with higher ERPT are Transport and Communications.

**Table 5:** Exchange Rate Pass-Through onto CPI and components before and after the break point: Error Correction, with Least Squares, Iceland 2003–2019*

	(1)	(2)	(3)	(4)
	Period One	Period Two	Period One	Period Two
	Short Term	Short Term	Long Term	Long Term
Aggregate CPI	0.129	0.050	0.194	0.075
Food and non-alcoholic beverages	0.202	0.066	0.275	0.090
Alcoholic beverages and tobacco	0.108	0.034	0.151	0.048
Clothing and footwear	0.258	0.128	0.137	0.068
Housing, water, electricity, gas, and other fuels	−0.0002	−0.0895	−0.0004	−0.2043
Furnishing and household equipment	0.293	0.091	0.391	0.121
Health	0.084	−0.029	0.086	−0.030
Transport	0.227	0.333	0.276	0.406
Communications	0.004	0.037	0.014	0.120
Recreation and culture	0.126	0.058	0.220	0.100
Educational services	−0.029	−0.052	−0.045	−0.081
Hotels, cafés and restaurants	0.084	−0.059	0.106	−0.075
Miscellaneous goods and services	0.060	0.020	0.093	0.030

*Period One corresponds to the estimated coefficient previous to the break point in 2009q1, and *Period Two* is after of the break point (includes it). *Short Term* is the contemporaneous coefficient of the exchange rate pass-through and *Long Term* is the estimated infinite sum.

To summarize, there is evidence that starting in 2009–2010 there was an important decline in the pass-through coefficient for the CPI. These results are consistent with the idea that around that time the CBI introduced changes in policy that were credible and effective.

## Nature of Shocks, Further Disaggregation, Robustness, and Other Extensions

### Nature of the Shocks and Pass-through

An important question raised by Forbes et al. (2018), is whether the pass-through coefficient is affected by the nature of the shocks that hit the economy. According to these authors, the pass-through coefficient will be different if the economy is subject to real shocks than if it is affected by monetary (or financial) shocks. In order to investigate this issue, we re-estimated a series of error correction equations with interactive terms for real exogenous shocks, defined as changes in the terms of trade, and for foreign financial shocks defined as the first difference in the Euro zone short-term interest rates. In this case, the estimated equations had the following form:

17 $$ {\pi}_t={\alpha}_0+{\alpha}_1\ \hat{E_t}+{\alpha}_2\ {\pi}_{t-1}+{\alpha}_3\ \Delta {\pi}_{t-1}+{\beta}_1\ {z}_t+\phi \kern0.5em \hat{E_t}\ {S}_t+{\omega}_{t.} $$

Where *S*_*t*_ denotes the shock in question (terms of trade or world interest rate). The short run pass-through will now be equal to (*α*_1_ + *ϕ S*_*t*_). That is, to the extent that the interactive term is statistically significant, the actual pass-through will depend on sign and size of the shocks, and on the new estimated parameter *ϕ*.

The results are presented in Table 6, for aggregate CPI inflation. In the analysis and interpretation that follow we concentrate on the third regression, where both interactive coefficients are included at the same time. As may be seen, the interactions are significant at conventional levels.

**Table 6:** Exchange Rate Pass-Through and Nature of Shocks: Iceland, Aggregate CPI, 2003–2019

**Variables**	(1)	(2)	(3)
NEER (% change)	0.105***	0.0876***	0.0861***
	(0.019)	(0.021)	(0.020)
NEER(% change)*foreign interest rate (change)	0.0592*		0.128***
	(0.031)		(0.042)
NEER(% change) * ToT (% change)		−0.138	−1.533**
		(0.493)	(0.644)
Price Index (% change) = 1 Lag,	0.492***	0.493***	0.495***
	(0.112)	(0.115)	(0.108)
Price Index (% change) = 1 Diff & 1 Lag,	−0.246**	−0.181	−0.277**
	(0.116)	(0.115)	(0.112)
Production Price Index (foreign)	0.235**	0.268***	0.273***
	(0.095)	(0.100)	(0.093)
Constant	0.00412**	0.00395**	0.00380**
	(0.002)	(0.002)	(0.002)
Observations	64	64	64
R-squared	0.6	0.575	0.636

Standard errors in parentheses

*** p < 0.01, ** p < 0.05, * p < 0.1

Consider first the results for the CPI inflation equation, in the presence of global financial shocks. In this case the pass-through coefficient will be equal to:

$$ ERPT={\alpha}_1+\phi \Delta  {r}^{EZ}, $$

where ∆*r*^*EZ*^ is the shock to the Eurozone short term interest rates and *ϕ* is the point estimate of the coefficient, 0.128. This means that if there is a positive shock to foreign interest rates equal to 50 basis points, the short run ERPT will be equal to 0.1501 (0.0861 + 0.128 × 0.50). This estimated pass-through coefficient is higher than the “clean” estimate of 0.0861, corresponding to the case when both global interest rates and terms of trade are in “equilibrium,” defined as the time when ∆*r*^*EZ*^ = 0 and ∆ % *ToT* = 0 (where ∆ % *ToT* is a terms of trade shock). However, it is still the case that the pass-through to CPI inflation is low.

We now turn to the case of real (terms of trade) shocks. Consider an improvement in Iceland’s terms of trade of 3%.^24^ In this case the point estimate of the short run ERPT for CPI inflation is a very low 0.0401 (0.0861–1.533 × 0.03). It is important to notice that the interpretation of the results in Table 6 are symmetrical to positive and negative external shocks. This means that if the terms of trade are reduced by 3%, the point estimate of the aggregate price index short-term pass-through coefficient will be equal to 0.132, indicating a faster pass-through.

As a further step in the analysis we decomposed both the real and financial shocks into positive and negative. The purpose of this decomposition is to determine if the response is symmetric to the sign of the disturbances. The results, not reported, but available upon request, indicate that there is no difference in the coefficient for positive and negative shocks.

### Further Disaggregation: A 65 Indexes Estimation

**Fig. 4:**
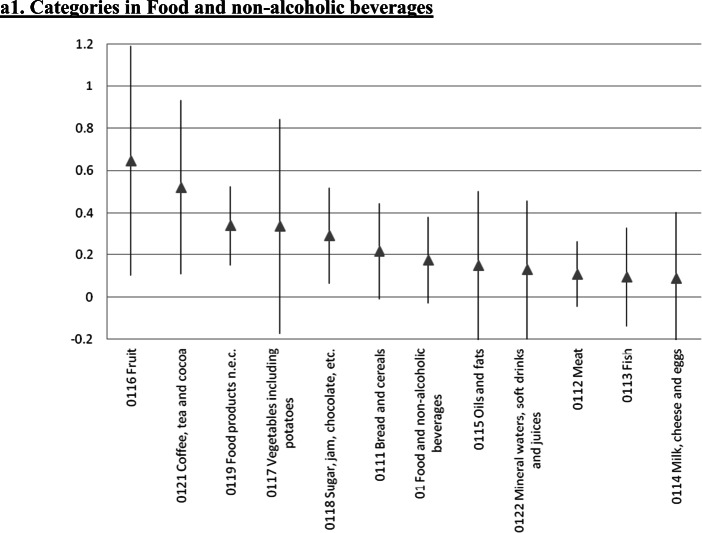

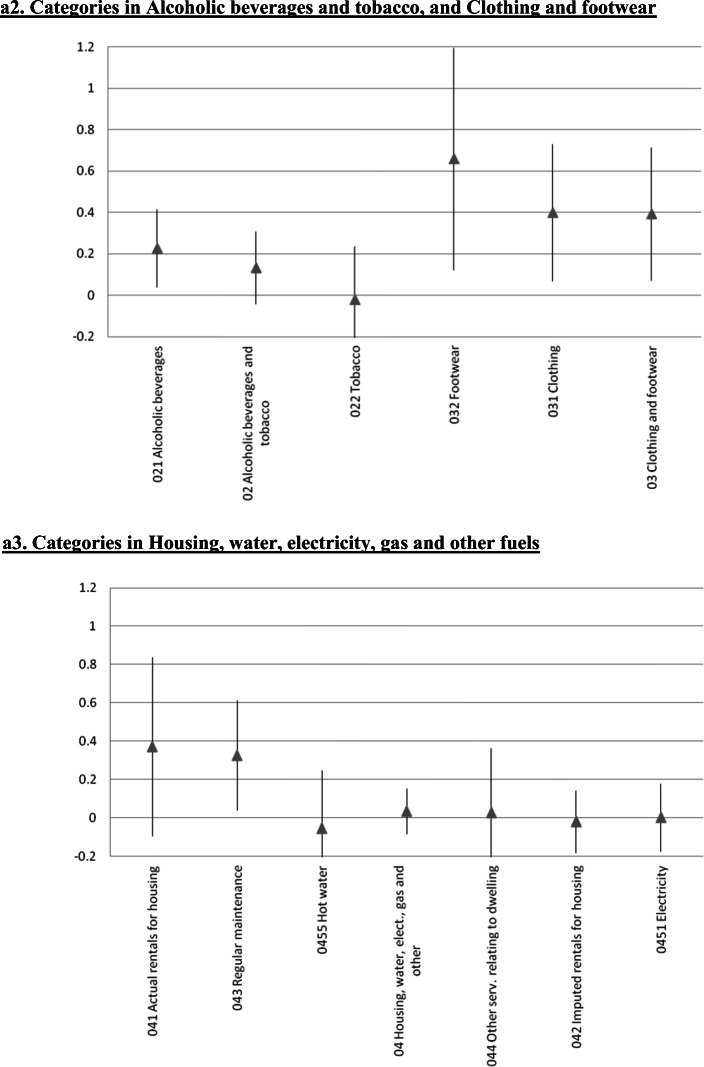

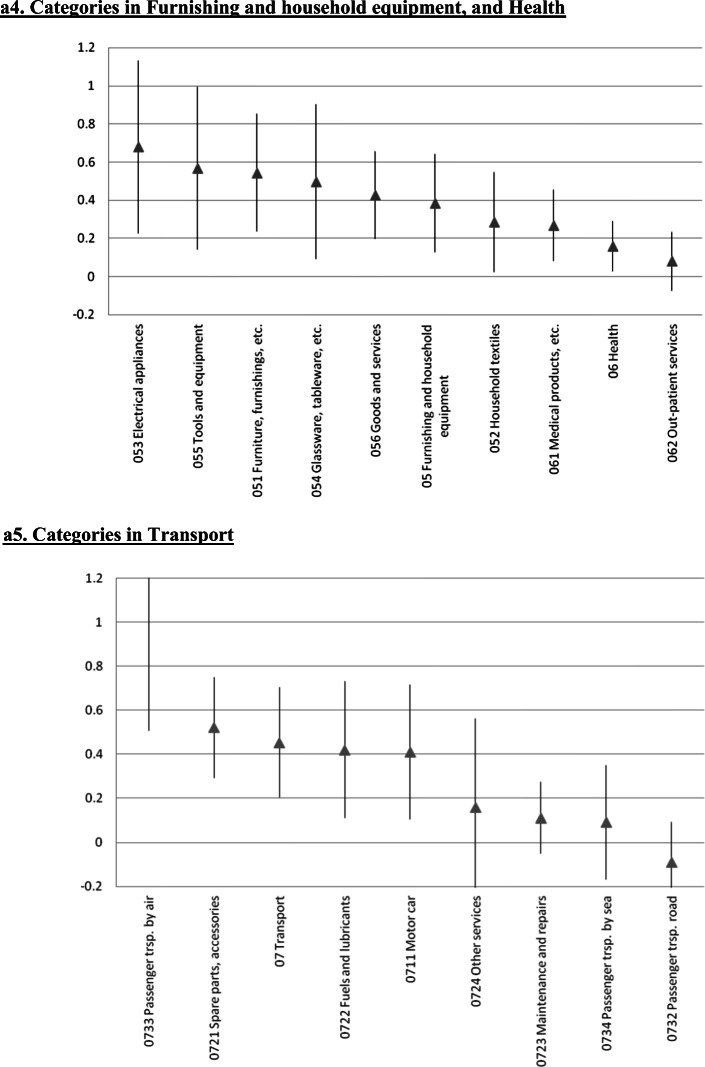

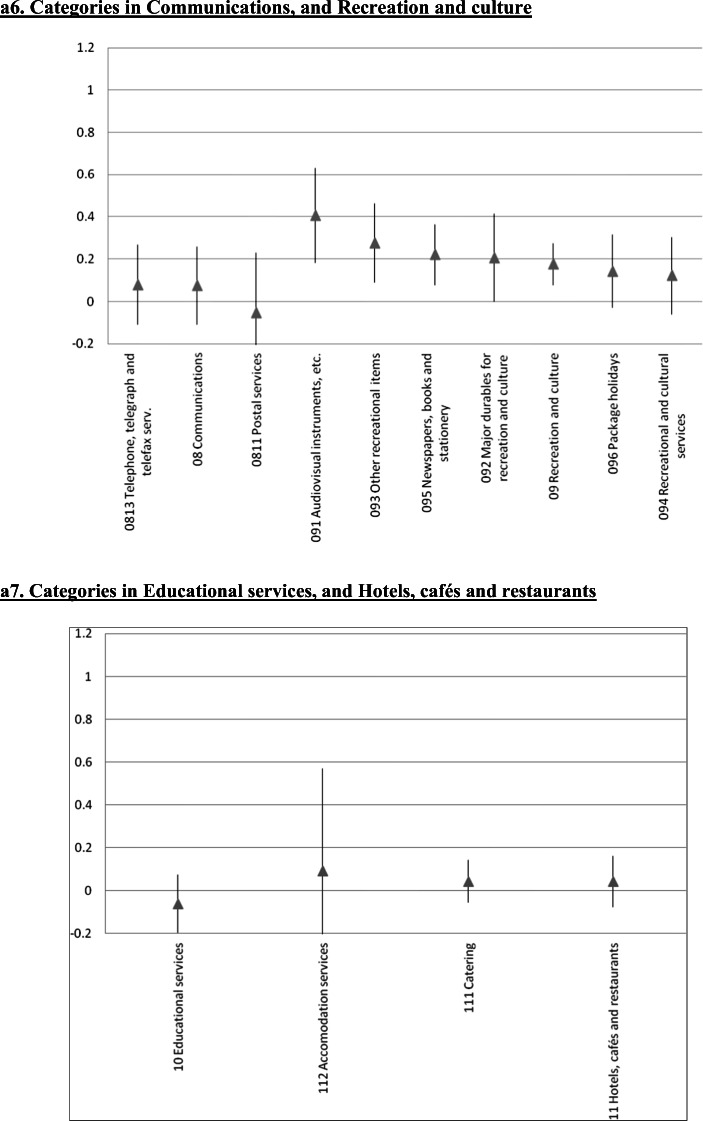

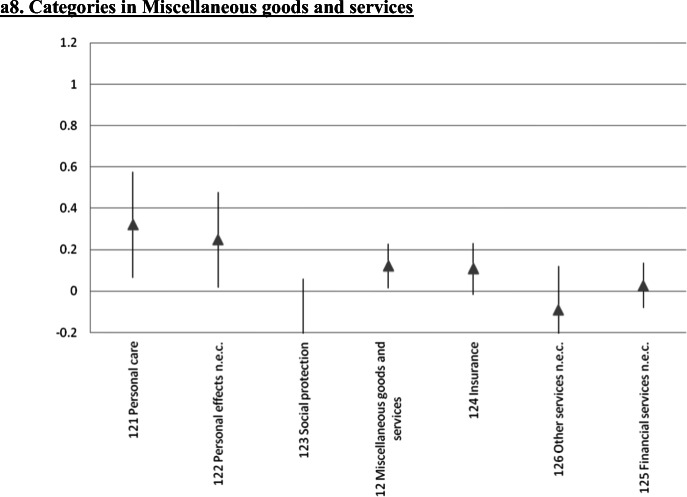
Exchange Rate Pass-Through onto CPI categories: Error Correction, Instrumental variables, Iceland 2003–2019, a1. Categories in Food and non-alcoholic beverages. a2. Categories in Alcoholic beverages and tobacco, and Clothing and footwear, a3. Categories in Housing, water, electricity, gas and other fuels. a4. Categories in Furnishing and household equipment, and Health. a5. Categories in Transport. a6. Categories in Communications, and Recreation and culture. a7. Categories in Educational services, and Hotels, cafés and restaurants. a8. Categories in Miscellaneous goods and services. The gray triangle at the center corresponds to the estimated coefficient following the same methodology as Table 1. Bar length corresponds to 95% confidence interval

We also estimated the error correction model at a significantly more disaggregated level. We focused on 65 of the 71 very detailed subcategories considered by Iceland’s statistical authorities for the CPI.^25^ The results for the short run ERPT coefficients are in Fig. 4, where each panel includes the components of the various sub-indexes analyzed in the preceding Sections. In these figures we present the point estimate as well as the 95% confidence interval. Not surprisingly, at this level of disaggregation the estimates are not very precise. Still, the main messages/conclusions from the previous Sections carry forward. Although there are differences in the ERPT coefficient within sub-indexes, it is still the case that tradable goods have higher ERPTs than nontradable ones.

### Robustness and Other Extensions

The results reported above were subject to a number of robustness tests. Overall, the results are sturdy to estimation method, sample, removal of outliers, different specifications of the error correction model, and alternative parametrization of the SVAR restrictions. In particular, the following issues are worth discussing briefly:

- Cook’s Distance tests were used to detect observations with “undue” influence. Outlier observations were excluded. The main results, however, were maintained.
- In addition to using a quarterly frequency, the equations discussed above were also estimated using monthly data. The estimated coefficients were less precise, as monthly data contains significantly more noise. However, the most important findings, in the sense that the pass-through coefficient for CPI inflation is rather low, and that different sectors have different coefficients is maintained. The ordering of ERPT coefficients is also maintained. The other results, in terms of breakpoints and interactions also hold, but, as pointed out, are less precise than with quarterly data.
- We analyzed in great detail the sensitivity of the structural VARs to the restrictions imposed on the structural matrices to convert VAR residuals into orthogonal structural innovations. As expected changes in the covariance structure affected the estimates. However, the most important aspect of the results, in the sense that there is a significant difference between the pass-through onto the aggregate CPI is maintained. In Table 7 we report a particular sensitivity analysis on the identifying restrictions of this structural matrix.
- We estimated the error correction models using different specifications, with additional covariates, including changes in interest rates and the cyclical component of GDP. The results, presented in Edwards and Cabezas (2021) in the Appendix C (Tables C.1 and C.2) broadly confirm those obtained in the body of this paper.
- In order to investigate further the role of structural breaks we also analyzed relative price volatility using Markov processes with switching variances. In line with our results from the Bai-Perron estimates, we found that there was a structural break in volatility in 2010. The data indicates the presence of two regimes: high variance (2003–2009) and low variance (2010 to 2019).
- As a way of testing the validity of our approach, we estimated error correction regressions and SVARs for eight “imported goods” price indexes collected by the Icelandic authorities. These indexes are constructed as the international price of each good category multiplied by the exchange rate: ${P}_{jt}={E}_t{P}_{jt}^{\ast }$. Given that Iceland is a small open economy, the estimation of our equations using the *P*_*jt*_ from these price indexes should result in both short and long-term pass-through coefficients not significantly different from one. Our analysis confirms this hypothesis, providing support to the empirical strategy that we have followed. Results and data are available on request.

**Table 7:** Sensitivity analysis of Covariance Restriction for estimation of SVAR for aggregate CPI*

Cov(*π*,NEER)	Short term	Long term
−0.02	0.108	0.193
−0.01	0.087	0.177
−0.005	0.078	0.169
0	0.07	0.161
0.005	0.061	0.153
0.01	0.052	0.144
0.02	0.032	0.122

*See text for details

## Concluding Remarks

In this paper we have used a unique data set for Iceland to investigate some often neglected aspects of the *exchange rate pass-through* problem. In particular, we have analyzed in great detail whether the pass-through coefficient differs across different categories of goods. We are particularly interested in three specific questions. First, is the ERPT significantly higher for tradables than for nontradables? A difference in the ERPT across these two categories of goods is essential for the nominal exchange rate to play an accommodative role in the face of shocks that require RER adjustments. There is evidence in the literature that in some countries that have gone through a major currency crisis (similar to the one in Iceland in 2008) the coefficient across goods is not significantly different. Second, we are interested in analyzing whether there was a structural break in the pass-through coefficient around the time the Central Bank of Iceland reformed its policy framework and strengthened its flexible inflation targeting (what has been called *inflation targeting plus*). Our model indicates that if the credibility of monetary policy increases, the exchange rate pass-through coefficient will decline. And third, we are interested in analyzing whether the pass-through depends on the nature of the shocks affecting the economy (real vs. financial).

We addressed these issues by using two estimation techniques: instrumental variables on error correction models, and VARs. The results obtained indicate that during the period under study the ERPT in Iceland was in the short run of nearly 0.15 and in the long term of about 0.23. It was significantly different across categories of goods; the range of sectorial long-run ERPT goes from zero to 0.72 (Table 2). It was substantially higher for tradable than for nontradable goods. We also found that there was a structural break in the pass-through coefficient in 2009. For the headline inflation, the coefficient declined significantly in the short run and in the long run, indicating that around that time there was an improvement in the degree of credibility of monetary policy. We also detected breakpoints in the majority of the components of the CPI. Finally, our analysis indicates that the size of the coefficients was affected by the nature of the shocks (financial or real). Our results are robust to the estimation technique and the specification of the equations.

## Appendix 1. Data definition and sources

**Consumer price index (CPI), aggregate and sub-indexes**: Consumer price aggregate and price categories. Index base 100 in March 1997. The quarterly data used is obtained as the mean of the three corresponding month. It starts in 2002q3 until 2019q1, and so it is base time period for the estimations that are involved. The variables used are the log difference.

The twelve price sub-indexes are: (1) Food and non-alcoholic beverages; (2) Alcoholic beverages and tobacco; (3) Clothing and footwear; (4) Housing, water, electricity, gas and other fuels; (5) Furnishing and household equipment; (6) Health; (7) Transport; (8) Communications; (9) Recreation and culture; (10) Educational services; (11) Hotels, cafés and restaurants; (12) Miscellaneous goods and services.

**Source:** Statistics Iceland.

**Nominal effective exchange rate****:** Corresponds to an index of nominal exchange rate weighted by imports. Index base 1 in 2005. Data used is at quarterly level obtained as the mean of the three corresponding month. The variable used is the log difference.

**Source:** Central Bank Iceland*.

**Production Price Index, trade weighted:** Foreign producer price index in manufacturing sector for domestic markets abroad (2015 = 100). Weights used are the same as in BIS for effective exchange rate that are based on trade. The variable used is the log difference.

**Source:** OECD Data and BIS.

**GDP - Euro Area:** This indicator is based on real GDP (also called GDP at constant prices or GDP in volume). The numbers are also adjusted for seasonal influences. The indicator is the log difference of volume index.

**Source:** OECD Data.

**Short term interest rate - Euro Area:** Short-term interest rates are based on three-month money market rates where available. Typical standardised names are “money market rate” and “treasury bill rate”. The variable used is in levels or the first difference depending the case.

**Source:** OECD Data.

**GDP – Iceland:** This indicator is based on real GDP (also called GDP at constant prices or GDP in volume). The numbers are also adjusted for seasonal influences. The indicator is the log difference of volume index.

**Source:** OECD Data.

**GDP cyclical component – Iceland:** This indicator is based on logarithm of real GDP and uses Hodrick-Prescott filter with smoothing parameter 1600 to recover cyclical component.

**Source:** OECD Data.

**GDP trend component – Iceland:** This indicator is based on logarithm of real GDP and uses Hodrick-Prescott filter with smoothing parameter 1600 to recover the trend. It is used the difference of this variable.

**Source:** OECD Data.

**Short term interest rate – Iceland:** Short-term interest rates are based on three-month money market rates where available. Typical standardised names are “money market rate” and “treasury bill rate”. The variable used is the first difference.

**Source:** OECD Data.

**Terms of trade:** We construct this index as the ratio of export over import prices multiplied by 100. The variable used is the log difference of the index.

**Source:** Statistics Iceland.

## Footnotes

1 ^I^ If the real exchange rate does not change sufficiently, adjustment will have to take place primarily through “expenditures reducing,” rather than through “expenditure switching.” This may result in significant unemployment costs.

2 There is an extensive and important literature on pass-through. A thorough review is beyond the scope of this paper. Some recent contributions include the literature that emphasizes the importance of identifying underlying shocks to assess the transmission of exchange rate movements to consumer prices (see Comunale and Kunovac 2017; Forbes et al. 2018; Corbo and Di Casola 2018; Ha et al. 2020, and An et al. 2020). In addition, some authors have explored the changing nature of the exchange rate pass-through with time series models (see De Souza et al. 2013; Erden and Ozkan 2015; Cunningham et al. 2017; Jašová et al. 2019). There is a growing literature that analyzes micro data on firm pricing decisions showing that firm performance and firm size (Berman et al. 2012), imported intermediate inputs in addition to market shares (Amiti et al. 2014), producers operating under incomplete information in a market (Garetto 2016), and the share of trade invoiced in foreign currencies and the dollar (Boz et al. 2020) affect the degree of pass-through.

3 See, for example, Edwards (2007). That study used the consumer price index (CPI) as a proxy for nontradables and the producer price index (PPI) as a proxy for tradables. That is, it didn’t have the granularity of the current study.

4 On the crisis see Jónsson (2009, 2019), Jónsson and Sigurgeirsson (2017), Aliber and Zoega (2019), and the various publications by the Central Bank of Iceland (CBI). On inflation dynamics and expectations in Iceland, see Pétursson (2020). A survey analysis of price setting behavior of Icelandic firms, with a special focus on ERPT is Ólafsson et al. (2011), with an updated survey reported in Central Bank of Iceland (2019).

5 For greater details, see Edwards (2018). See, also, Jónsson (2009) and Jónsson and Sigurgeirsson (2017), the various publications by the Central Bank of Iceland, and in particular Pétursson (2018, 2019). Also the essays in Aliber and Zoega (2019).

6 This was just before the demise of the Scandinavian Monetary Union.

7 On October 30, 2020, the Minister of Finance and Economic affairs published a list of the (very few) remaining controls (see Iceland 2020). A useful reference for the history of exchange rate arrangements in Iceland is Guðmundsson et al. (2000).

8 See Jónsson (2009) and Jónsson and Sigurgeirsson (2017), for illuminating and in depth analyses. During the last few years – since the 2012 change in policy stance -- the CBI has intervened occasionally in the foreign exchange market. After the pandemic the number of interventions increased. This has happened in a number of countries, as documented by the IMF on its site on Covid-19 responses.

9 For references on the crisis see Jónsson and Sigurgeirsson, (2017), and the various reports by the IMF. In particular, the Article IV Consultation documents. Also, Aliber and Zoega (2019).

10 One could quibble about “Transport.” However, the fact that operational costs are mostly related to the cost of fuel makes it a (mostly) tradable sector.

11 See also Burstein et al. (2007) and the literature cited herein. See, also, the literature cited in footnote 2.

12 Since this is a small country, it faces given (exogenous) international prices of importables. However, what matters for measuring inflation is the domestic price, once the goods are retailed to the public. It is perfectly possible that retail operations are subject to some degree of monopolistic competition, which justifies considering a “pricing to market” perspective, and a markup approach.

13 A recent box in the CBI Monetary Bulletin estimates trade elasticities (Central Bank of Iceland, 2015). The long-run ERPT to import prices is also found to be essentially −1.

14 See, however, the discussion on full backward looking indexation below.

15 Edwards (1993, 2011). In the more general formulation, additional terms will appear in these equations, including, in particular, international inflation.

16 In Edwards and Cabezas (2021) in the Appendix B.2 is provided a unit root test of the main variables used in these estimations. The results indicate that null hypothesis of the presence of unit root is rejected.

17 We computed F-tests for weak exogeneity of instruments, as well as Sargan tests for over identification. The results indicate that for nine out of the twelve CPI components the null of weak instruments is rejected with *p* < 0.15.

18 See above for our classification of the 12 sub-indexes into tradable and nontradable.

19 When alternative structures were used, the results were similar.

20 Additionally, when we impose reasonable restrictions on the covariance between changes in inflation and exchange rates (covariance between inflation and exchange rate changes between 0.005 and − 0.005) the results from the structural VARs are very similar to those obtained from the Cholesky decomposition. Given this similarity in the results, we only provide figures for the IRF obtained using the Cholesky decomposition.

21 CBI (2017, pp. 34–36).

22 The paper by Gagnon and Ihrig (2004) finds evidence that greater monetary policy credibility leads to lower ERPT.

23 Although the instrumental variables estimates are less precise, they tell a similar story.

24 Taking the data for the period under consideration, an improvement (or a decline) of ± 5% corresponds, approximately, to the 90th percentile.

25 We excluded 6 redundant categories that overlapped in a significant way with some of the indexes already considered.
